# A Critical Role of δ-Opioid Receptor in Anti-microglial Activation Under Stress

**DOI:** 10.3389/fnagi.2022.847386

**Published:** 2022-05-19

**Authors:** Yuan Xu, Feng Zhi, Ya Peng, Jiahao Mao, Gianfranco Balboni, Yilin Yang, Ying Xia

**Affiliations:** ^1^Clinical Medical Research Center, The Third Affiliated Hospital of Soochow University, Changzhou, China; ^2^Department of Neurosurgery, The First People’s Hospital of Changzhou, Changzhou, China; ^3^Shanghai Key Laboratory of Acupuncture Mechanism and Acupoint Function, Fudan University, Shanghai, China; ^4^Department of Life and Environment Sciences, University of Cagliari, Cagliari, Italy

**Keywords:** microglia, injury, inflammation, δ-opioid receptor, lipopolysaccharide, hypoxia

## Abstract

Microglia are involved in the regulation of cerebral homeostasis and pathogen confrontation. There is, however, evidence showing that excessive microglia activation is implicated in various age-related cerebral diseases. On the other hand, microglia may experience complex changes of polarization in pathological insults, i.e., from a proinflammatory M1 to an anti-inflammatory M2 phenotype, which differentially contribute to the exacerbation or alleviation of cellular injury. Remolding the phenotype of microglia or inhibiting the excessive activation of microglia seems to be a promising approach against neurodegenerative pathologies. Since δ-opioid receptor (DOR) activation exhibits a strong protective capacity against various neuronal injuries, especially the hypoxic/ischemic injury, we asked if the DOR-induced neuroprotection is associated with its effect on microglia. We explored this fundamental issue by using pharmacological and genetic approaches in the BV2 cell line, a general type of microglial cells. The results showed that DOR expression significantly increased in the activated microglial M2 phenotype, but slightly decreased in the microglial M1 phenotype. Hypoxia induced dual polarizations of BV2 cells with an increase in DOR expression. Administration of a specific DOR agonist, UFP-512, largely inhibited lipopolysaccharide (LPS) or hypoxia-induced microglial M1 activation and inflammatory activity with high concentrations of UFP-512 being effective to reverse the interleukin-4 (IL4)-induced microglial activation. Consistent with these observations, inhibiting DOR or knocking-down DOR promoted the excessive activation of BV2 cells in both M1 and M2 directions, while DOR overexpression did the opposite. Furthermore, the PC12 cells exposed to the conditioned medium of BV2 cells treated by UFP-512 grew better than those treated directly with UFP-512 under LPS or hypoxic insults. DOR inhibitor naltrindole could block all the effects of DOR activation. The medium from the BV2 cells with DOR knock-down decreased the viability of PC12 cell, while the medium from the BV2 cells with DOR overexpression largely attenuated LPS or hypoxic injury in the PC12 cells. These first data suggest a close linkage between DOR expression/function and microglial polarization and a critical role of DOR in negative controlling microglial activation. Our work provides a novel clue for new protective strategies against neurodegenerative pathophysiology through DOR-mediated regulation of microglia.

## Introduction

Microglia are the resident immune cells originated from mesodermal progenitors in the yolk sac (Wieghofer et al., 2015), and they function as the first line of defense within the central nervous system (CNS) (Prinz et al., 2019). More recently, with the development of imaging, genetics and single-cell sequencing technologies, emerging evidence has suggested that microglia play more complex roles in both physiological and pathological conditions.

Microglia are a major mediator of neuroinflammations by producing cytokines, chemokines and iNOS and inducing a broad spectrum of cellular responses (Colonna and Butovsky, 2017; Voet et al., 2019). They express many important immune receptors (Colonna and Butovsky, 2017). For instance, microglia express toll-like receptors (TLRs) to detect pathogens, and express NOD-like receptors such as NLRP3 inflammasome to induce neuroinflammations. Several endotoxin and pathogens can bind to the TLR4 on the microglia surface and activate the pro-inflammatory activities of microglia (Nair et al., 2019). An increase in the pro-inflammatory cytokines and chemokines on one hand promotes the massive infiltration of leukocytes within the CNS and thus impairing blood-brain barrier (BBB). On the other hand, pro-inflammatory cytokines such as TNF-α and IL- 1β initiate the apoptotic program of neurons by activating caspase and mitogen- activated protein kinase (MAPK) signaling (Gualtierotti et al., 2017; Shabab et al., 2017). ROS and iNOS produced by microglia function as an enhancer for neuroinflammation. They trigger the inflammatory events by activating several genes involved in the regulation of inflammatory cascades and thus generate more cytotoxic agents implicated in neuronal death (Shabab et al., 2017; Simpson and Oliver, 2020; Ivan et al., 2021). The excessive production of ROS also led to mitochondrial dysfunction and tissue injury (Shabab et al., 2017).

Moreover, excessive activations of microglia are implicated in several age-related cerebral diseases with selective changes in gene expression and significant alterations in cellular morphology, motility, migration, and proliferation (Wu et al., 2020). For example, stroke, which is a leading cause of death and disability worldwide with ischemic stroke accounting for >80% of the all cases (Krishnamurthi et al., 2020). Once ischemic or hypoxic injury occurs, microglia migrate toward the lesion site subsequently and produce various of inflammatory cytokines and cytotoxin to induce inflammations. On the other hand, microglia drive the phagocytosis of apoptotic neurons and debris and secret growth factors and anti-inflammatory cytokines to promote neurogenesis and tissue repair (Ma et al., 2017). The dynamic microglia activation can be protective at the beginning, but destructive in prolonged stresses. The role of microglia largely depends on its different polarization in response to different pathological stages and cellular microenvironment during the injury (Ma et al., 2017; Zhang et al., 2021). Therefore, elucidating the mechanisms of microglial activation, and exploring pharmaceutical ways to modify microglia transformation or inhibit excessive microglia activation are of great significance in terms of new therapies for refractory age-related cerebral diseases.

δ-opioid receptor (DOR) is a major family member of opioid receptors. It is widely distributed in the cortex and caudate putamen in the brain (Xia and Haddad, 1991, 2001; Xia, 2015; Pellissier et al., 2018). Besides the involvement of DOR in pain modulation, strong evidences support its neuroprotective role in several diseases including ischemic/hypoxic injury (Feng et al., 2012; Chao et al., 2016; Xu Y. et al., 2016; Chen et al., 2020). It is a chief regulator of ionic homeostasis (Chao et al., 2016; Chen et al., 2020), neuronal transmission (Zhang and Pan, 2012; Yokota et al., 2016; Birdsong et al., 2019) and inflammations (DiCello et al., 2018; Geng et al., 2020). However, most of these studies were conducted on neurons or neuron-like cells *in vivo* and *in vitro*. There is only limited information on the distributions and functions of DOR in microglia. Shrivastava et al. (2017) reported that μ-opioid receptor and DOR differentially regulate microglial inflammation in response to neonatal ethanol in the hypothalamus of rats, and they found that DOR activation promoted microglial secretion of anti-inflammatory cytokines and suppressed ethanol induced microglial inflammatory activation. More recently, a research group examined the role of DOR in lipopolysaccharide (LPS)-induced microglial activation, they observed that DOR activation could inhibit LPS-induced inflammatory response and improved microglia survival by switching microglia to a beneficial phenotype (Cheng et al., 2021).

Following these clues, our study aimed to specify the role of DOR in microglia, not only in normal condition, but also under hypoxic/ischemic stress. By using pharmacological and genetic approaches, we examined microglial DOR expression profile in different states, the effects of DOR on microglia polarizations, inflammatory events and the interactions of DOR and microglia in neuroprotection. Our results strongly suggest a direct involvement of DOR in inflammatory regulation via inhibiting microglia activation under LPS and hypoxic injury.

## Materials and Methods

### Chemicals and Reagents

UFP-512, a highly specific DOR agonist, was synthesized by our group (Polo et al., 2019; Xu et al., 2019, 2020b; Chen et al., 2020). Naltrindole hydrochloride, a highly selective DOR antagonist was purchased from MedChemExpress (Cat: HY-101177, NJ, United States). Anti-DOR antibody (dilution for immunofluorescence: 1:200; dilution for Western blot: 1:1000) and Anti- Iba1 antibody (dilution for immunofluorescence: 1:100) were purchased from Abcam (Cat: ab176324, ab283319, Shanghai, China). Anti- MHCII antibody (dilution for Western blot: 1:1000) was purchased from Santa Cruze Biotechnology (Cat: sc-32247, TX, United States). Dulbecco’s Modified Eagle Medium (DMEM) for cell culture, fetal bovine serum (FBS), Goat anti- Rabbit IgG (H + L) highly cross- absorbed secondary antibody, Goat anti- mouse IgG (H + L) highly cross-adsorbed secondary antibody for immunofluorescence, TRlzol reagent and BCA protein assay kit were all purchased from ThermoFisher Scientific (Cat: 11995040, 10082147, A21429, A32723, 15596018, 1859078, Shanghai, China). Anti-β-actin antibody (dilution for Western blot: 1:2,000) was purchased from Cell Signaling Technology (Cat: 3700S, CO, United States). Cell counting kit-8 (CCK8), Hoechst 33258 staining solution, Immunostaining permeabilization buffer with triton X- 100, 4% paraformaldehyde fix solution, and Arginase antibody (dilution for Western blot: 1:1,000) were all purchased from Beyotime. Co (Cat: C0039, C1017, P0096, AF1381, Shanghai, China). Hiscript^®^ II Q RT supermix for qPCR was purchased from Vazyme Biotech Co (Cat: R223-01, Nanjing, China). SYBR select master mix was purchased from applied biosystems (Cat: 4472913, CA, United States). 10% ExpressCast PAGE Gel Preparation kit for Western blot was purchased from New Cell & Molecular Biotech Co. (Cat: P2012, Suzhou, China). LPS was purchased from sigma-Aldrich (Cat: L2630, Shanghai, China). IL4 was purchase from Peprotech (Cat: 200-04, NJ, United States).

### Cell Cultures and Groupings

Mouse microglial BV2 cell line was obtained from National Infrastructure of Cell Line Resource (NICR), Beijing, China (Cat: 1101MOU-PUMC000063). The highly differentiated rat PC-12 cell line was purchased from the Type Culture Collection of the Chinese Academy of Sciences, Shanghai, China. Both BV2 cells and PC12 cells were cultured in DMEM containing 10% FBS, and randomly subjected to normoxic, LPS, IL4 and hypoxic treatment. The cells in the normoxic group were maintained in a humidified incubator with 5% CO_2_ at 37°C. To induce microglial M1/M2 polarization, BV2 cells were exposed to LPS (5 μg/ml) or IL4 (20 ng/ml) for 24 h, respectively. To induce hypoxia, cells were moved to a hypoxic chamber (Galaxy 48R, New Brunswick, Edison, NJ, United States) with 1% O_2_ levels for 48 h. DOR specific agonist UFP-512 (5 μM) and DOR antagonist naltrindole (1 μM) were used to treat BV2 cells or PC12 cells for 48 h as we previously described (Xu Y. et al., 2016; Xu et al., 2019, 2020b).

### Immunofluorescence Staining

The BV2 cells were seeded on microscope cover glass in a 12-well plate. Each well was fixed with 4% paraformaldehyde fix solution for 20 min, and then rinsed by 1x PBS for three times. Immunostaining permeabilization buffer with triton X-100 were used to promote cells’ permeabilization. After washing with 1x PBS for another three times, the cells were incubated in a 1x PBST containing 3% FBS for 1 h at room temperature and then transferred to a buffer that contained DOR antibody (1:200) and Iba antibody (1:100) overnight at 4°C. Secondary antibody against rabbit IgG (4 ug/ml), conjugated with fluorescent dyes Alexa- Fluor 555 and secondary antibody against mouse IgG (4 μg/ml), conjugated with fluorescent dyes Alexa-Fluor 488 were used to detect the DOR and Iba expression, respectively. Hoechst staining solution was used to detect the cell nucleus. Incubations without primary antibody were set as negative controls. The fluorescence staining cells were finally visualized and photographed using the Leica TCS-SP2 confocal scanning microscope (Leica, Heidelberg, Germany).

### Western Blotting

Proteins were extracted from the cells using the RIPA buffer containing 0.5% 100 Mm PMSF, 0.1% protease inhibitors and 1% phosphatase inhibitors (Cat: 4693132001, 4906837001, Roche, Basel, Switzerland). The protein concentration was determined using the BCA protein assay kit. Equal amounts of protein samples were loaded into 7.5–15% SDS-PAGE gel to separate, and the separated proteins were transferred to polyvinylidenedifluoride (PVDF) membranes. The protein membranes were blocked by 1x PBST with 5% skim milk or 5% BSA for 1 h at room temperature, and probed with respective primary antibodies overnight at 4°C. The secondary antibodies were used to detect the different protein expression, and the bands were visualized by the Western lightening Chemiluminescence Reagent Plus (Perkin-Elmer, Boston, MA, United States). The band densities were analyzed using ImageJ software.

### Lentivirus Infection

The lentiviral plasmids expressing shRNAs against DOR were obtained from GENECHEM, Shanghai, China. To knockdown DOR, three plasmids (103885-1, 103886-1, 103887-2) expressing three different shRNA sequences were applied, and a negative control plasmid (CON207) was set. To overexpress DOR, one plasmid called 71788-1 was used, and negative control (CON294) was set as well. The BV2 cells were seeded on 6- well plate with the concentrations of 2 × 10^5^ cells per well for 24 h. HiTransG A (GENECHEM, Shanghai, China) was added together with lentiviral plasmids in 10% FBS DMEM to promote the infection efficiency (MOI = 10). After 8 h infection, the medium containing plasmids and HiTransG A was removed, and the infected cells were cultured in fresh medium for another 48 h. Two microgram per milliliter puromycin was used to screen the infected cells for 48 h. Then PCR and western blotting were used to quantify the DOR level.

### mRNA Quantification

To measure the changes in the DOR mRNA level, and evaluate the inflammatory situation in the cellular environments, total RNA was extracted and purified using Trizol reagent following the instruction. Then the extracted RNA (1 μg) was used as the template to generate cDNA. RT-PCR (Applied Biosystems, CA, United States) was performed to quantify the mRNA relative content. The primers designed are listed as follows:

**Table:** 

DOR mRNA primers	F: 5′ GGCTGTGCTCTCCATTGACT 3′
	R: 5′ GACACCTGAAGCCAAGACCC 3′
iNOS mRNA primers	F: 5′ CAGCTGGGCTGTACAAACCTT 3′
	R: 5′ CATTGGAAGTGAAGCGTTTCG 3′
TNF-α mRNA primers	F: 5′ CAAGGGACAAGGCTGCCCCG 3′
	R: 5′ GCAGGGGCTCTTGACGGCAG 3′
CD206 mRNA primers	F: 5′ TTCGGTGGACTGTGGACGAGCA 3′
	R: 5′ ATAAGCCACCTGCCACTCCGGT 3′
IL-10 mRNA primers	F: 5′ GGTTGCCAAGCCTTATCGGA 3′
	R: 5′ ACCTGCTCCACTGCCTTGCT 3′
TGF-β mRNA primers	F: 5′ GGAGCCACAAACCCCGCCTC 3′
	R: 5′ GCCAGCAGGTCCGAGGGGAGA 3′
GAPDH mRNA primers	F: 5′ GCCAAGGCTGTGGGCAAGGT 3′
	R: 5′ TCTCCAGGCGGCACGTCAGA 3′

### Cell Viability Assay

Cell viability was measured by CCK8 kit. Exponentially growing cells were seeded at 5,000 cells/well in a 96-well plate. A blank control was set with the well no cells but containing culture medium. The PC12 cells were treated with fresh culture medium with different drugs, the supernatant collected from BV2 cultures, or the PC12 cells were co-cultured with BV2 cells. After appropriate duration of treatments, the original medium was removed and 100 ml of fresh culture medium was added. Ten microliters of CCK8 reagent per well was applied to the cells for 2 h for complete reaction. The absorbance was measured at the wavelength of 450 nm using a microplate reader (Biotek, VT, United States).

### Statistical Analysis

**FIGURE 1:**
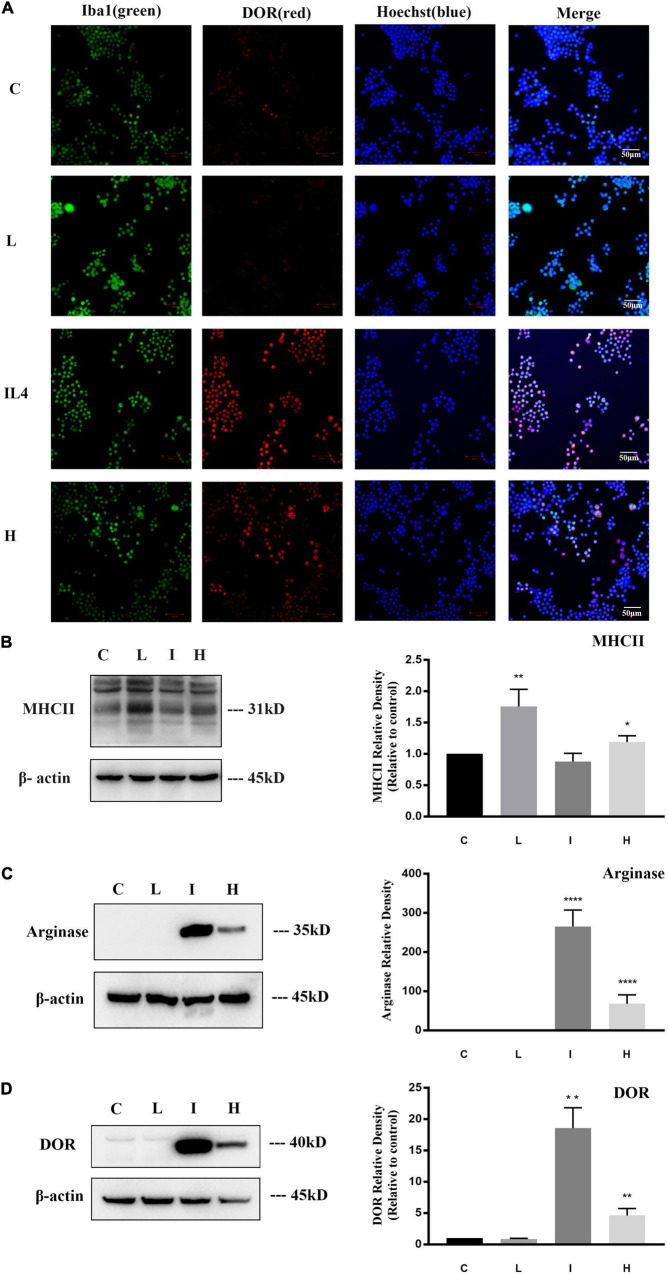
δ-opioid receptor (DOR) expression and distribution in BV2 cells. The experiments were conducted on BV2 cells (*N* = 3 in each group). C: normal control. L: LPS. I: IL4. H: 1% O_2_ hypoxia. **(A)** Representative immunofluorescent images of BV2 cells exposed to normal, LPS, IL4, or hypoxic environment. The merge images merged Iba1 (green), DOR (red), and Hoechst (blue) images in each group. Note that the immunofluorescence staining showed that DOR maintained a mild expression level under normal condition and LPS insult, but the red fluorescence labeling DOR significantly enhanced after 24 h of IL4 treatments. Hypoxia induced both high-expressed DOR and low-expressed DOR BV2 cells. **(B)** Differential MHCII expression in BV2 cells under physiological, LPS, IL4, or hypoxic condition. *p < 0.05, **p < 0.01 vs. C. Note that LPS significantly upregulated MHCII expression, while hypoxic insult also induced a slight increase in MHCII protein in BV2 cells. **(C)** Differential Arginase expression in BV2 cells under physiological, LPS, IL4, or hypoxic condition. ****p < 0.0001 vs. C. Note that arginase is difficult to detect under normal condition or LPS insult. IL4 caused a sharp increase of arginase, and hypoxia also led to an upregulation of Arginase protein. **(D)** Alterations of DOR density in BV2 cells under physiological, LPS, IL4, or hypoxic condition. **p < 0.01 vs. C. Note that DOR density kept a low level under normal condition and LPS insult, but significantly increased after exposure to IL4 or hypoxia.

**FIGURE 2:**
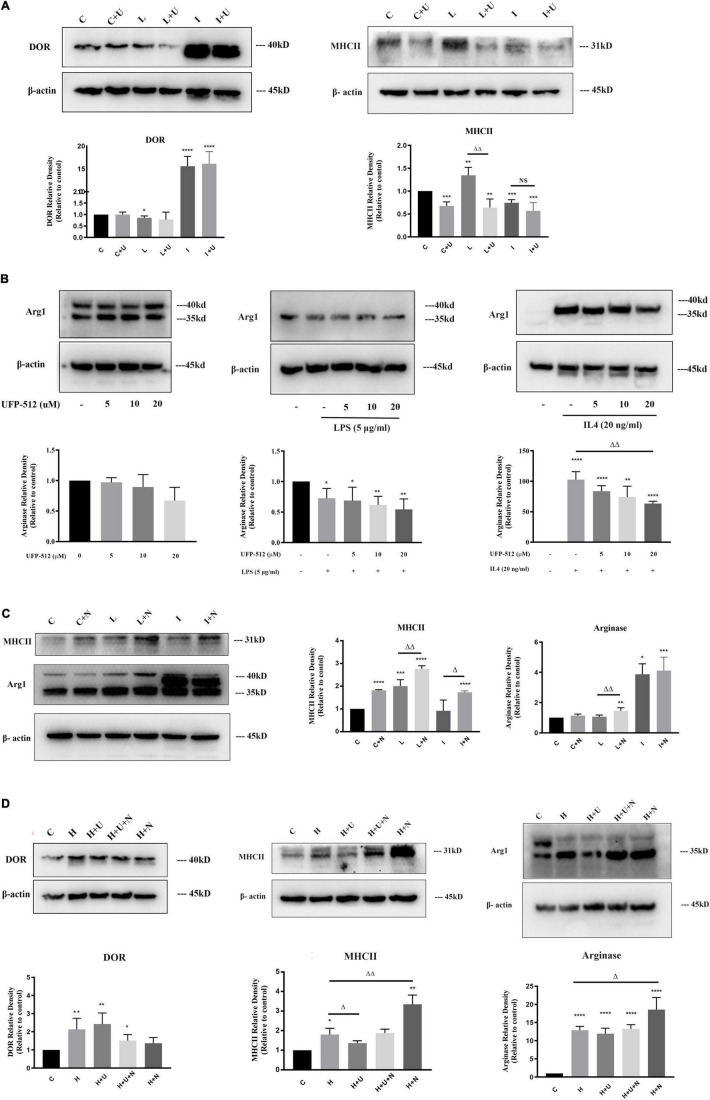
Effects of δ-opioid receptor (DOR) activation on microglial M1 transformation in LPS or hypoxic insult, and microglial M2 transformation under IL4 condition. The expression levels of DOR, MHCII and Arginase were examined by Western blot in BV2 cells at different states (*N* ≥ 3 in each group). C: control. C + U: DOR activation by UFP-512 under normal condition. C + N: DOR inhibition by naltrindole normal condition. L: LPS. L + U: UFP-512 plus LPS. L + N: naltrindole plus LPS. I: IL4. I + U: UFP-512 plus IL4. I + N: naltrindole plus IL4. H: hypoxia. H + U: DOR activation by UFP-512 under hypoxic insults. H + U + N: UFP-512 plus naltrindole under hypoxic insults. H + N: DOR inhibition by naltrindole under hypoxic insult. **(A)** DOR-induced inhibition of MHCII expression under normal condition and LPS insult. **p < 0.01, ***p < 0.001, ****p < 0.0001 vs. C; ^ΔΔ^*p* < 0.01 vs. L. Note that the administration of UFP-512 did not appreciably change the expression level of DOR in BV2 cells, but DOR activation significantly downregulated MHCII under normal condition and LPS insult. **(B)** Effect of DOR activation on arginase in BV2 cells. *p < 0.05, **p < 0.01, ****p < 0.0001 vs. C; ^ΔΔ^*p* < 0.01 vs. L. Note that 5–20 μM UFP-512 did not cause an appreciable change in arginase under normal condition and LPS insult, but the 20 μM of UFP-512 significantly reduced arginase protein in the BV2 cells exposed to IL4. **(C)** Effects of DOR inhibition on MHCII and arginase in BV2 cells. *p < 0.05, **p < 0.01, ***p < 0.001, ****p < 0.0001 vs. C; ^ΔΔ^*p* < 0.01 vs. L; ^Δ^*p* < 0.05 vs. I. Note that the application of naltrindole largely upregulated MCHII under normal condition, LPS insult and IL4 insult. DOR inhibition significantly increased arginase protein under LPS insult. **(D)** Effects of DOR activation on BV2 activation in hypoxic stress. The BV2 cells were exposed to hypoxic environment, UFP-512 and naltrindole were simultaneously or separately applied to these cells for 48 h. *p < 0.05, **p < 0.01, ****p < 0.0001 vs. C; ^Δ^*p* < 0.05, ^ΔΔ^*p* < 0.01 vs. H. Note that hypoxia induced an upregulation of DOR in BV2 cells, while DOR activation or inhibition did not change the level of DOR in a major way. The applications of UFP-512 largely inhibited MHCII expression under hypoxic insults, while the addition of naltrindole reversed this effect. The BV2 cells treated with naltrindole alone under hypoxic insults showed a significant increase in both MHCII and arginase.

**FIGURE 3:**
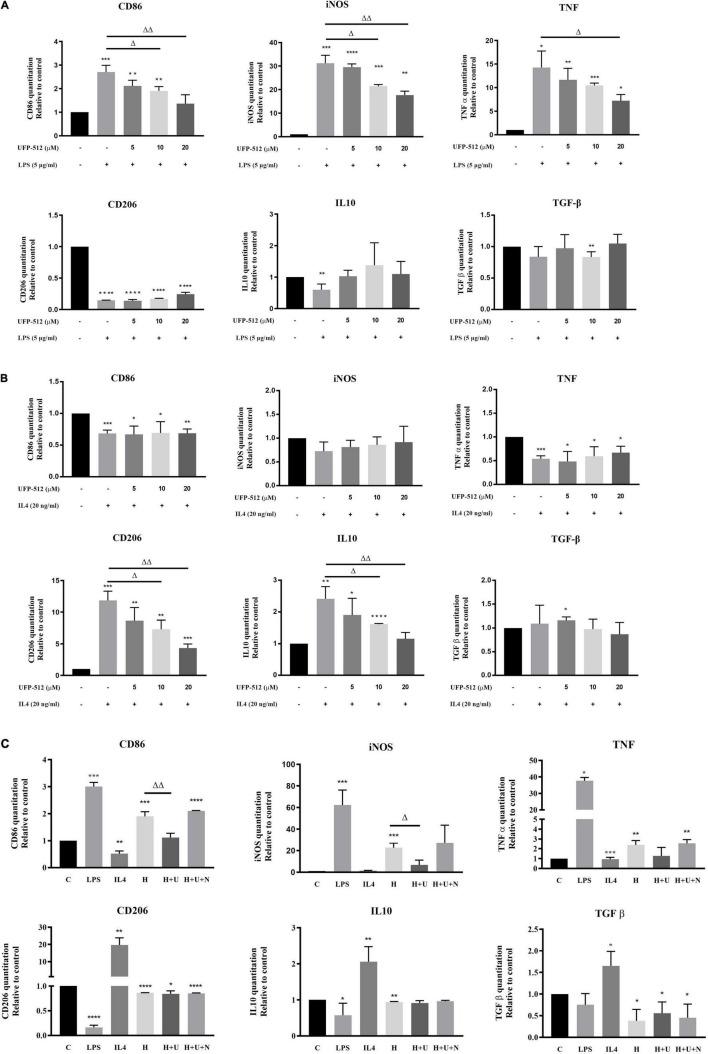
δ-opioid receptor (DOR) regulation of inflammatory cytokines and anti-inflammatory cytokines under LPS, IL4, or hypoxic condition. The mRNA levels of CD86, iNOS, TNF-α, CD206, IL10, and TGF-β were measured by RT-PCR in BV2 cells under LPS/hypoxic insults (*N* ≥ 3 in each group). **(A)** Effects of DOR activation on inflammatory events under LPS insult. *p < 0.05, **p < 0.01, ***p < 0.001, ****p < 0.0001 vs. C; ^Δ^*p* < 0.05, ^ΔΔ^*p* < 0.01 vs. L. Note that 10–20 μM of UFP-512 significantly inhibited the M1 type shift of BV2 cells under LPS insult, as well as downregulating iNOS and TNF. **(B)** DOR-mediated suppressions of anti-inflammatory events in IL4-treated BV2 cells. *p < 0.05, **p < 0.01, ***p < 0.001, ****p < 0.0001 vs. C; ^Δ^*p* < 0.05, ^ΔΔ^*p* < 0.01 vs. I. Note that IL4 caused a significant increase in M2 phenotype marker CD206 and anti-inflammatory cytokines IL-10 and TGF- β. Following the increase of UFP-512 concentration, the IL4- mediated anti-inflammatory events were attenuated. **(C)** Effects of DOR activation on inflammatory events under hypoxic stress. *p < 0.05, **p < 0.01, ***p < 0.001, ****p < 0.0001 vs. C; ^Δ^*p* < 0.05, ^ΔΔ^*p* < 0.01 vs. H. Note that the hypoxia-induced upregulation of CD86, iNOS and TNF-α were inhibited by DOR activation with UFP-512, while the addition of naltrindole reversed these effects.

**FIGURE 4:**
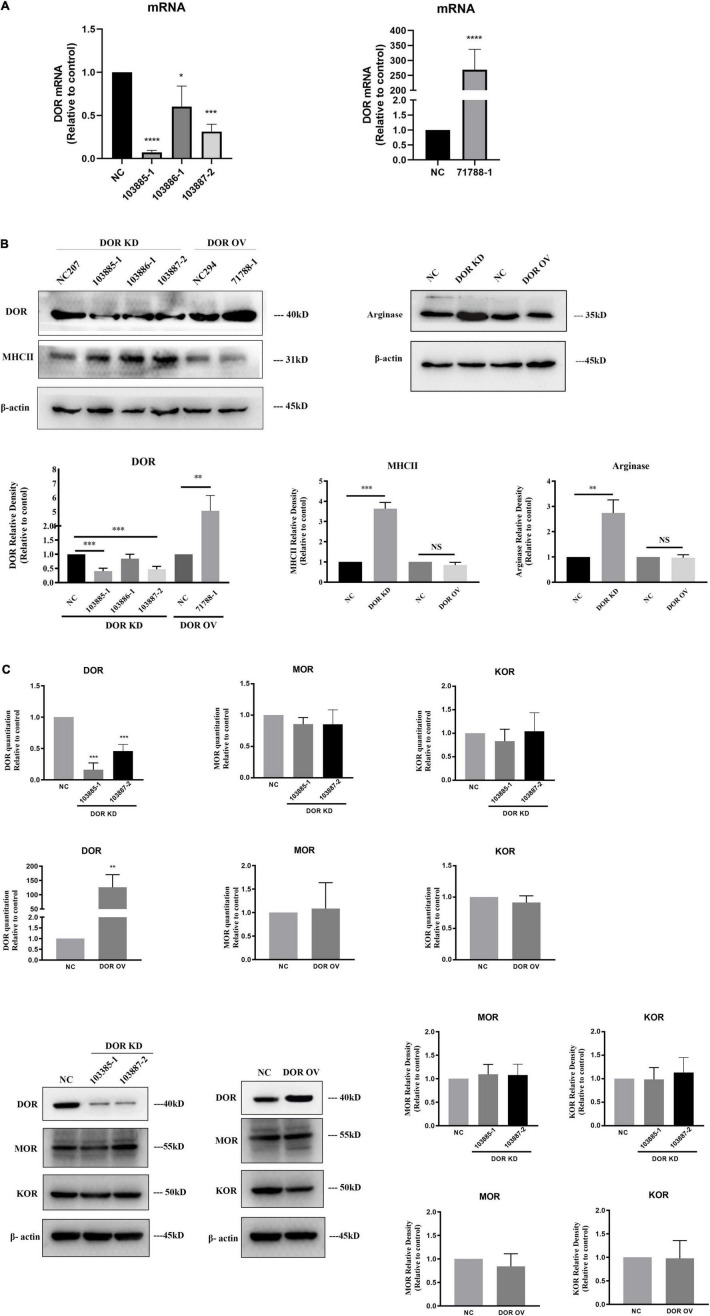
A critical role of δ-opioid receptor (DOR) for anti-microglial activation. NC, negative control; DOR KD, DOR knockdown; DOR OV, DOR overexpression. **(A)** RT-PCR evaluation of the efficiency of plasmids infection. *p < 0.05, ***p < 0.001, ****p < 0.0001 vs. NC. Note that the DOR knockdown shRNA plasmids infections significantly reduced DOR mRNA expression in BV2 cells, especially the 103885-1 and 103887-2. The BV2 cells infected with 71788-1 plasmids showed folds increases in DOR mRNA compared to the negative control. **(B)** DOR knockdown or overexpression-induced alterations in MHCII and arginase protein expression. **p < 0.01, ***p < 0.001 vs. NC. Note that DOR knockdown caused an upregulation of both MHCII and arginase, while DOR overexpression did not seriously affect MHCII and arginase expression. **(C)** Effects of DOR knockdown or overexpression on MOR and KOR of BV2 cells. **p < 0.01, ***p < 0.001 vs. NC. Note that 103885-1 and 103887-2, the DOR knockdown shRNA plasmids significantly reduced DOR mRNA expression in BV2 cells. The BV2 cells infected with 71788-1 plasmids showed folds increases in DOR mRNA compared to the negative control. Neither DOR knockdown nor overexpression caused any appreciable change in MOR and DOR expression by RT-PCR or Western blot measurements.

**FIGURE 5:**
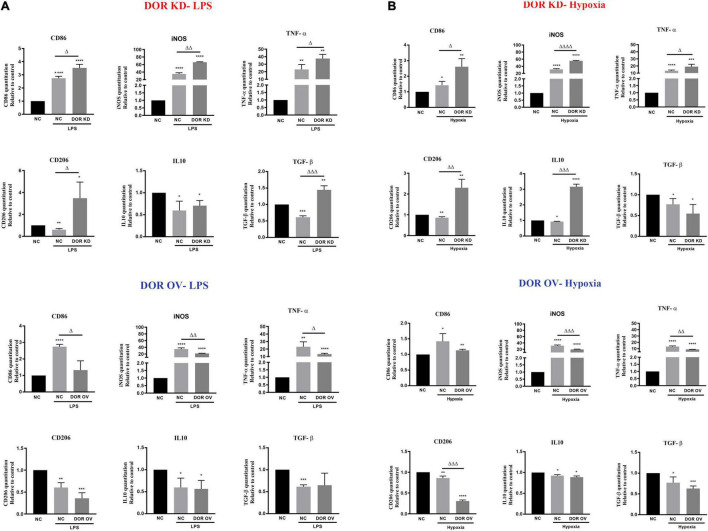
δ-opioid receptor (DOR) knockdown and overexpression induced opposite effects on microglia-mediated inflammatory events. **(A)** Effects of DOR knockdown and overexpression on inflammatory cytokines under LPS insult. *p < 0.05, **p < 0.01, ***p < 0.001, ****p < 0.0001 vs. NC; ^Δ^*p* < 0.05, ^ΔΔ^*p* < 0.01, ^ΔΔΔ^*p* < 0.001 vs. NC-LPS. Note that DOR knockdown aggravated the inflammatory situation induced by LPS, and it also promoted the anti-inflammatory events to fight against LPS injury. DOR overexpression largely downregulated LPS-induced generations of CD86, iNOS and TNF-α, while showed an inappreciable effect on CD206, IL-10 and TGF-β. **(B)** Effects of DOR knockdown or overexpression on inflammatory cytokines under hypoxic stress. *p < 0.05, **p < 0.01, ***p < 0.001, ****p < 0.0001 vs. NC; ^Δ^*p* < 0.05, ^ΔΔ^*p* < 0.01, ^ΔΔΔ^*p* < 0.001, ^ΔΔΔΔ^*p* < 0.0001 vs. NC-Hypoxia. Note that DOR knockdown in BV2 cells significantly increased both the inflammatory cytokines and anti-inflammatory cytokines level under hypoxic stress. DOR overexpression in BV2 cells suppressed the inflammatory events, and downregulated M2 phenotype marker CD206 as well.

**FIGURE 6:**
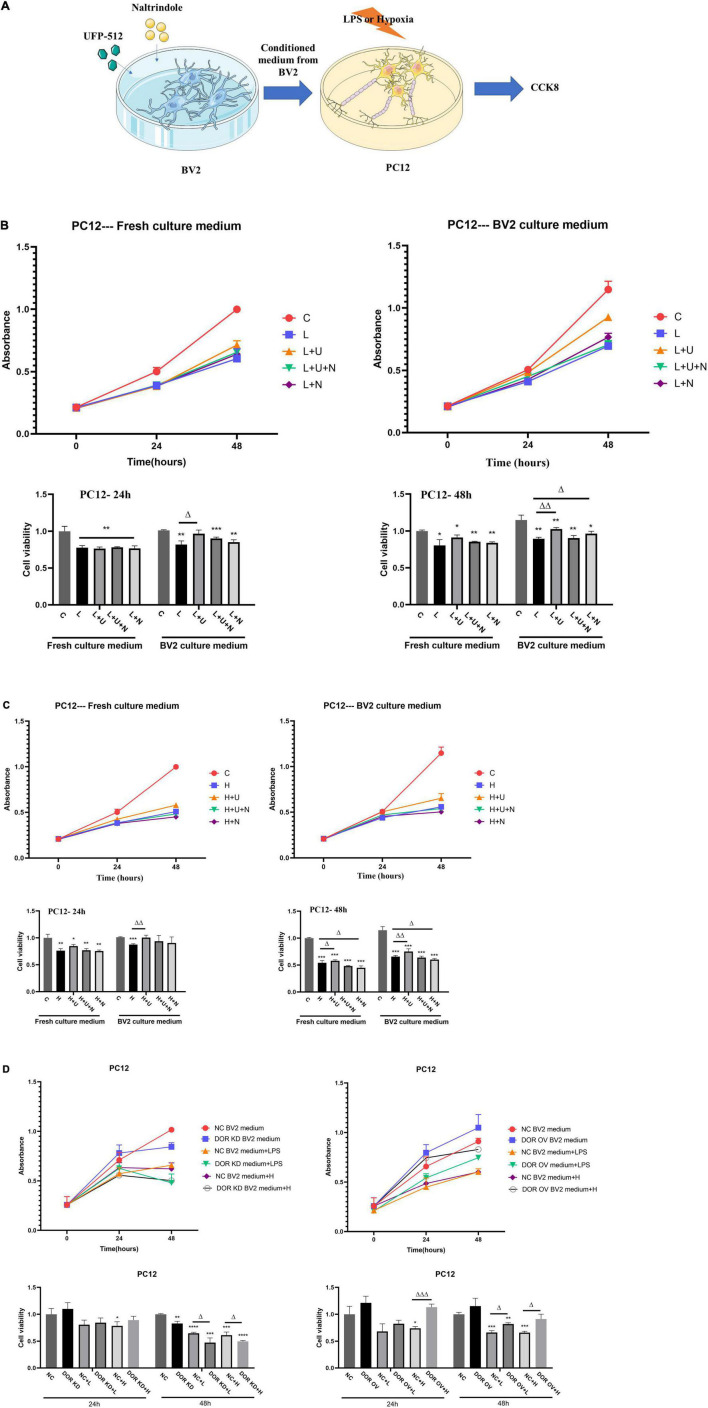
Effects of conditioned medium from BV2 cells with δ-opioid receptor (DOR) activation/inhibition on PC12 cell viability in LPS or hypoxic stress. C: the control. L: LPS. L + U: direct UFP-512 treatment or medium from UFP-512 treated BV2 cells under LPS insult. L + U + N: direct treatment of UFP-512 plus naltrindole or medium from UFP-512 plus naltrindole-treated BV2 cells under LPS insult. L + N: PC12 cells were directly treated with naltrindole or cultured with the conditioned medium from naltrindole-treated BV2 cells in LPS insult. H: hypoxia. H + U: UFP-512 plus hypoxia. H + U + N: UFP-512 plus naltrindole under hypoxia. H + N: hypoxia plus naltrindole. NC: negative control. DOR KD: PC12 cells were cultured in the medium from BV2 cells with DOR knockdown. OV: PC12 cells were cultured in the medium from BV2 cells with DOR overexpression. + L: PC12 was cultured in BV2 medium and then exposed to LPS insult. +H: PC12 was cultured in BV2 medium and then exposed to hypoxic insults. **(A)** Schematic diagram showing CCK8 measurements of highly differentiated PC12 cells after being treated with conditioned medium from BV2 culture. **(B)** Cell viability and DOR-mediated alterations in cell proliferation under LPS insult. *p < 0.05, **p < 0.01, ***p < 0.001 vs. C; ^Δ^ *p* < 0.05, ^Δ^ ^Δ^ *p* < 0.01 vs. L. Note that LPS caused a significant decrease in cell viability, applications of the conditioned medium from UFP-512 treated BV2 cells largely increased the cell viability at both 24-h timepoint and 48-h timepoint. Using the conditioned medium from naltrindole-treated BV2 cells also promoted cell proliferations under LPS injury after 48 h incubation. **(C)** Effect of DOR activation on cell viability under hypoxic stress. *p < 0.05, **p < 0.01, ***p < 0.001 vs. C; ^Δ^ *p*0.05, ^Δ^ ^Δ^ *p* < 0.01 vs. H. Note that DOR activation with UFP-512 could directly increase PC12 cell viability in hypoxic stress after 48-h incubation, while DOR inhibition induced an opposite effect. When the conditioned medium from the BV2 cells were applied to the culture of PC12 cells, the UFP-512 induced neuroprotection became more evident, and appeared earlier. The cell viability of the PC12 cells cultured with conditioned medium from naltrindole treated BV2 cells still decreased. **(D)** The alterations in PC12 cell viability by manipulating DOR in BV2 cells. *p < 0.05, **p < 0.01, ***p < 0.001, ****p < 0.0001 vs. NC; ^Δ^ *p* < 0.05, ^Δ^ ^Δ^ ^Δ^*p* < 0.001 vs. NC + L or NC + H. Note that the PC12 cells cultured in the medium from BV2 cells with DOR knockdown showed a significant decrease in cell viability after 48-h LPS exposure or hypoxic exposure. To the contrary, PC12 cells treated with the medium from BV2 cells with DOR overexpression showed a large increase in cell viability under the stresses, especially the hypoxic stress.

All data are presented as means ± SD. Each independent experiment was performed at least three times. The number (n) of conducted experiments is indicated in figure captions. One-way analysis for variance (ANOVA) followed by Bonferroni’s multiple comparison tests was used to analyze statistical significance for MHCII, arginase and DOR expression profile, as well as the inflammatory-related cytokines (Figures 1–5) (Prism 5, GraphPad Software, CA, United States). Two-way ANOVA was used to analyze the main effects of the BV2-conditioned medium on PC12 cell viability as compared to the viability of PC12 cells cultured by fresh culture medium, and to compare the differences in DOR function within the groups (Figures 6B–D).

## Results

### δ-Opioid Receptor Expression Profile Was Closely Associated With the Microglial Polarization

Firstly, we investigated the expression of DOR in microglia. As shown in Figure 1A, BV2 cells were identified by Iba1 immune-staining, a marker for both resting and activated microglia with a green fluorescent, and DOR immune-staining with a red fluorescent. The green fluorescent labeled iba1 could be detected in all the groups. A weak red fluorescence signal labeling DOR was detected in the control group and the group treated with LPS, while the fluorescence signals were largely enhanced in the group treated with IL4 (20 ng/ml) (Figure 1A). These data provided the first evidence that DOR expression is differentially regulated in response to different microglial phenotypes.

To further confirm this DOR expression profile, we evaluated the protein level of DOR in different microglial states by Western blot. MHCII, a M1 activated microglia marker, and arginase, a M2 activated microglia marker, were used to identify microglial phenotype. The results showed that LPS caused a significant increase of MHCII expression in the BV2 cells (^**^p < 0.01, Figure 1B), while IL4 remarkably upregulated Arginase (^****^p < 0.0001, Figure 1C), suggesting a classic transformation of BV2 cells to M1 and M2 phenotypes, respectively. Then, we measured DOR expression in BV2 cells with the M0, M1 and M2 phenotypes. As Figure 1D depicted, DOR maintained a low density in the cells with no treatment and those treated with LPS. However, following the stimulation of IL4, DOR expression greatly increased (^**^p < 0.01, Figure 1D). The Western blot data were very consistent with the immunofluorescent results, suggesting that the expression of DOR is closely associated with the states of microglia.

### Hypoxia Induced a Biphasic Activation of Microglia With an Upregulation of δ-Opioid Receptor

Previous studies reported the complex role of microglial cells in ischemic stroke pathobiology (Guruswamy and ElAli, 2017), we therefore studied the effect of hypoxic stress on microglia. The BV2 cells were exposed to 1% O_2_ for 48 h and then the polarizations of microglia and alterations of DOR expression were investigated. Dual immunofluorescence staining showed that the DOR signals with a red fluorescent were enhanced in some activated microglia, but were still weak in the remaining microglia (Figure 1A).

Moreover, we measured MHCII and arginase to determine the degree of activation in the hypoxia-exposed BV2 cells. Compared to the cells in physiological condition, the hypoxia-exposed cells showed a significant increase in both MHCII (*p < 0.05, Figure 1B) and arginase (^****^p < 0.0001, Figure 1C), suggesting that hypoxia induced a biphasic polarization of microglia. We also observed an increased DOR expression in the hypoxia-exposed BV2 cells with the expression profile being positively associated with microglia activation (^**^p < 0.01, Figure 1D).

### δ-Opioid Receptor Activation Inhibited Microglial Inflammatory Transformation in Lipopolysaccharide or Hypoxic Stress

Next, we asked if DOR activation/inhibition alters the phenotypes and functions of microglia.

Firstly, 5 μg/ml LPS or 20 ng/ml IL4 were used to induce microglia polarization. DOR agonist UFP-512 (10 μM) was added together with LPS or IL4 to treat BV2 cells for 24 h. By comparing the alterations in MHCII and Arginase before and after DOR activation, we evaluated the involvement of DOR in microglial activations. Consistent with the results in the previous sections, DOR density kept in a low level in the control and LPS-treated BV2 cells, while IL4 largely increased DOR expression in the BV2 cells. The administration of DOR agonist UFP-512 did not cause an appreciable change in DOR expression in all the groups (Figure 2A). but led to a significant decrease in MHCII protein in the control group and LPS-treated group (with a 36.0% reduction in the control group, ^***^p < 0.001; and a 59.9% reduction in the LPS-treated group, ^ΔΔ^*p* < 0.01; Figure 2A). IL4-treated BV2 cells originally expressed a mild level of MHCII, activating DOR in these cells did not further reduce MHCII expression (Figure 2A).

We also measured the alterations in arginase following the concentration gradient of UFP-512 to evaluate the effects of DOR on microglial activation. As the Figure 2B showed, LPS caused a remarkable decrease in arginase (*p < 0.05). An increase in UFP-512 concentration did not appreciably change the level of arginase in both the control and LPS-treated groups (Figure 2B, left panel and middle pane). IL4 treatment remarkably upregulated Arginase expression as mentioned above, while DOR activation gradually down-regulated the level of arginase following the increase of UFP-512 concentration (^ΔΔ^*p* < 0.01, Figure 2B, right panel).

To further verify the effects of DOR on microglia activation, we used naltrindole (1 μM), a DOR antagonist, to treat BV2 cells under physiological condition, LPS condition and IL4 condition, respectively. As Figure 2C depicted, DOR inhibition by naltrindole significantly enhanced the M1 transformation of BV2 cells by upregulating MHCII protein in each condition (^****^p < 0.0001 vs. C; ^ΔΔ^*p* < 0.01 vs. L; ^Δ^*p* < 0.05 vs. I, Figure 2C). Meanwhile, naltrindole largely increased arginase expression in LPS-exposed BV2 cells (^ΔΔ^*p* < 0.01 vs. L, Figure 2C), suggesting that DOR inhibition and activation had opposite effects on microglia activation.

Since hypoxia induced bipolar activations of BV2 cells, we then investigated the effects of DOR on BV2 cells exposed to hypoxia. UFP-512 (10 μM) was used to activate DOR and naltrindole (1 μM), a DOR antagonist, was used to inhibit DOR activities. The cells were immediately exposed to hypoxic environment after treated by UFP-512 and/or naltrindole. Neither DOR activation nor inhibition changed DOR expression level in the BV2 cells seriously (Figure 2D, left panel). Notably, DOR activation remarkably reduced MHCII expression under hypoxic condition (^Δ^*p* < 0.05, Figure 2D, middle panel), while the simultaneous addition of naltrindole reversed the effects induced by UFP-512. The administration of naltrindole alone even caused a sharp increase of MHCII in the BV2 cells (^ΔΔ^*p* < 0.01, Figure 2D, middle panel). Moreover, the results showed that arginase level was not seriously changed by DOR activation (Figure 2D, right panel). Instead, DOR inhibition using naltrindole significantly upregulated arginase level in the BV2 cells exposed to hypoxia (^Δ^*p* < 0.05, Figure 2D, right panel).

All these data suggest that DOR activation could effectively inhibit the inflammatory polarizations of microglia under LPS or hypoxia, while the inhibition of DOR could amplify the inflammatory transformation. Our studies also provided the new information that the high concentrations of DOR agonist UFP-512 (20 μM) could also suppress the shift of BV2 cells to M2 phenotype, a phenotype responsible for repair, anti-inflammation and regeneration.

### δ-Opioid Receptor Was Involved in the Regulation of Microglial Inflammatory Events

Microglia are the main effectors in the inflammatory process of the CNS, and the microglia-mediated inflammatory events are largely depending on its different polarizations (Xu L. et al., 2016). Therefore, we evaluated the effects of DOR on microglia-mediated inflammatory events by measuring several immune markers, inflammatory cytokines and anti-inflammatory cytokines by RT-PCR. The data showed that the exposure to LPS significantly increased the levels of CD86, iNOS and TNF-α mRNA in the BV2 cells (*p < 0.05, ^***^p < 0.001 vs. C, Figure 3A, upper panel), while the administration of DOR agonist UFP-512 gradually inhibited their production with the increase in the concentration (^Δ^*p* < 0.05, ^ΔΔ^*p* < 0.01 vs. L, Figure 3A, upper panel). On the other hand, LPS caused a sharp reduction in CD206, a M2 phenotype marker, and a decrease in the level of IL-10 mRNA (^***^p < 0.001 vs. C, Figure 3A, bottom panel). When treating the cells with 5–20 μM of UFP-512 for 24 h, DOR activation did not appreciably affect these anti-inflammatory cytokines and markers as a whole (Figure 3A, bottom panel).

When BV2 cells were treated with IL4, the M2 marker CD206 and IL10 was remarkably increased (^**^p < 0.01, ^***^p < 0.001 vs. C, Figure 3B, bottom panel) with a decrease in M1 marker, CD86 and pro-inflammatory cytokine, TNF (^***^p < 0.001 vs. C, Figure 3B, upper panel). DOR activation largely reduced CD206 and IL10 mRNA levels (^Δ^*p* < 0.05, ^ΔΔ^*p* < 0.01 vs. I, Figure 3B, bottom panel), while did not change the levels of CD86, iNOS and TNF mRNA (Figure 3B, upper panel).

Finally, we asked whether hypoxic stress causes similar changes. Although it has been observed that hypoxia induced biphasic polarization of BV2 cells as stated above, 1% O_2_ hypoxia for 48 h led to a remarkable increase of CD86, iNOS and TNF with a decrease in CD206, IL10 and TGF- β (*p < 0.05, ^**^p < 0.01, ^***^p < 0.001 vs. C, Figure 3C), suggesting a tendency of pro-inflammatory transformation of microglia. DOR activation with UFP-512 (10 μM) significantly downregulated CD86 and inhibited the productions of iNOS, but showed no effect on TNF, CD206, IL10 and TGF-β (^Δ^*p* < 0.05, ^ΔΔ^*p* < 0.01 vs. H, Figure 3C). The DOR activation induced effects on CD86 and iNOS could be reversed by the addition of DOR antagonist naltrindole.

### δ-Opioid Receptor Played a Critical Role for Anti-microglial Activation

To clarify the direct effects of DOR on microglia, we used lentiviral plasmids expressing DOR shRNAs to infect the BV2 cells. As Figure 4 depicted, the plasmids 103385-1, 103886-1, and 103887-2 infections caused an 89.4% reduction (103885-1: ^****^p < 0.0001 vs. NC), 39.7% reduction (103886-1: *p < 0.05 vs. NC) and 63.7% reduction (103887-2: ^***^p < 0.001 vs. NC, Figure 4A, upper panel) in DOR mRNA, respectively, while plasmids 71788-1 infections led to 268 folds of increase in DOR mRNA compared to the negative control (^****^p < 0.0001 vs. NC, Figure 4A, bottom panel). The Western blot results were consistent with the trends of DOR mRNA changes (^**^p < 0.01, ^***^p < 0.001 vs. NC, Figure 4B), confirming the successful shRNA design and plasmids infection in this work.

Firstly, we asked whether the manipulation of DOR expression induced any change in other opioid receptors. We examined the expression of μ-opioid receptor (MOR) and κ-opioid receptor (KOR) in the BV2 cells with DOR knockdown or DOR overexpression. Compared to the negative control group, neither DOR knockdown nor DOR overexpression significantly affected MOR and KOR at the mRNA and protein levels in the BV2 cells (Figure 4C).

We then examined the alterations in MHCII and arginase before and after DOR knockdown or overexpression. The data showed that DOR knockdown significantly upregulated both MHCII (^***^p < 0.001 vs. NC) and Arginase proteins (^**^p < 0.01 vs. NC, Figure 4B), while DOR overexpression did not affect MHCII and arginase at all (Figure 4B).

The effects of DOR knockdown or overexpression on LPS- and hypoxia-induced inflammatory events were also investigated. As Figure 5A showed, DOR knockdown largely increased M1 marker CD86 and M2 marker CD206 under LPS insult (^Δ^*p* < 0.05 vs. NC-LPS). The inflammatory cytokines iNOS, TNF-α, and anti-inflammatory cytokines TGF- β were also significantly enhanced (^Δ^*p* < 0.05,^ΔΔ^ *p* < 0.01, ^ΔΔΔ^*p* < 0.001 vs. NC-LPS, Figure 5A). Compared to the performance of DOR knockdown under LPS insult, DOR overexpression remarkably downregulated the microglial M1 phenotype marker CD86 and inhibited the production of iNOS and TNF (^Δ^*p* < 0.05,^ΔΔ^ *p* < 0.01 vs. NC-LPS, Figure 5A), but had no effect on M2 microglial marker CD206, and two anti-inflammatory cytokines IL-10 and TGF- β.

When exposed to 1% O_2_ hypoxia, the cells infected with DOR knockdown plasmids showed a significant increase in both inflammatory cytokines and anti-inflammatory cytokines (^Δ^*p* < 0.05,^ΔΔ^ *p* < 0.01, ^ΔΔΔ^*p* < 0.001, ^ΔΔΔΔ^*p* < 0.0001 vs. NC-Hypoxia), while those infected with DOR overexpressing plasmids showed an attenuation in iNOS and TNF production, as well as a deprivations of M2 phenotype marker CD206 (^ΔΔ^*p* < 0.01, ^ΔΔΔ^*p* < 0.001 vs. NC-Hypoxia, Figure 5B). DOR overexpression had no effect on IL10 and TGF-β under hypoxia (Figure 5B)

### The Medium of BV2 Cells Treated by UFP-512 Attenuated Lipopolysaccharide- or Hypoxia-Induced Neuronal Injury

We have previously demonstrated DOR-mediated neuroprotection against hypoxic/ischemic injury (Xu Y. et al., 2016). To identify whether the DOR-mediated neuroprotection is relevant to DOR’s modulation of microglia, we treated the BV2 cells with 5 μM DOR agonist UFP-512 or 1 μM DOR antagonist naltrindole for 24 h, and then collected the BV2 medium for culturing highly differentiated PC12 cells, a neuron-like cells. The cell viability was measured using CCK8 every 24 h (Figure 6A). As shown in Figure 6B, the conditioned medium from the UFP-512 treated BV2 cells effectively protected the PC12 cells against LPS insult at both 24-h (^Δ^*p* < 0.05) and 48-h timepoints (^ΔΔ^*p* < 0.01, Figure 6B). Although a direct addition of UFP-512 to PC12 cells also caused an elevation of cell viability at 48-h timepoint, the increase was not statistically significant (Figure 6B). More interestingly, culturing the PC12 cells with the conditioned medium from the naltrindole-treated BV2 cells for 48 h under LPS insult also surprisingly increased the cell viability (^Δ^*p* < 0.05, Figure 6B). In contrast, the cell viability of the PC12 cells treated directly with naltrindole for 48 h mildly decreased (Figure 6B).

The same experiments were conducted under hypoxic condition. As shown in Figure 6C, DOR activation with UFP-512 for 48 h effectively protected PC12 cells from hypoxic injury with a significant increase in cell viability (^Δ^*p* < 0.05, Figure 6C), while DOR inhibition using naltrindole remarkably reduced PC12 cell viability and aggravated the hypoxic injury (^Δ^*p* < 0.05, Figure 6C). When using the culture medium from BV2 cells instead, we found that the DOR-mediated neuroprotection became more evident, and the protective effects appeared earlier, i.e., at the 24-h timepoint (^ΔΔ^*p* < 0.01, Figure 6C). The additions of naltrindole reversed the effects of UFP-512, while the conditioned medium from naltrindole-treated BV2 cells significantly decreased the cell viability of PC12 cells and aggravated the hypoxic injury as well.

We then examined the effects of the medium from the BV2 cells with DOR knockdown or overexpression on PC12 cells under normal condition, LPS insult and hypoxic stress. The results showed that the PC12 cells exposed to the medium from the BV2 cell with DOR knockdown for 24 h did not cause any appreciable change in cell viability compared to those exposed to the medium from the BV2 cell with negative control plasmids (Figure 6D, left panel). However, after 48-h exposure to normal, LPS or hypoxia condition, the PC12 cells cultured in the medium from the BV2 cell with DOR knockdown showed a significant decrease in cell viability (^**^p < 0.01 vs. NC, ^Δ^*p* < 0.05 vs. NC + L or NC + H, Figure 6D, left panel).

We also compared the alterations in cell viability between the PC12 cells cultured in the medium from the BV2 cell with negative control plasmids and those in the medium from the BV2 cell with DOR overexpression. The results showed that the viability of the PC12 cells cultured in the medium from BV2 cells with DOR overexpression was significantly improved under hypoxic stress at both 24- and 48-h time points (^ΔΔΔ^*p* < 0.001 vs. NC + H at 24 h; ^Δ^*p* < 0.05 vs. NC + H at 48 h, Figure 6D, right panel). Also, there was a major difference between the PC12 cells cultured in the medium from the BV2 cell with negative control plasmids and those in the medium from the BV2 cell with DOR overexpression under LPS insult for 48 h with the latter having a much better cell growth (^Δ^*p* < 0.05 vs. NC + L in 48 h, Figure 6D, right panel). All these data suggest that the DOR neuroprotection is mediated, at least partially, by DOR’s modulation of microglia.

## Discussion

We have made following interesting findings in the BV2 cell, an immortalized murine microglia model (Khan and Langmann, 2020; Wu et al., 2020; Cheng et al., 2021): (1) DOR maintained a low density in resting microglia and could be largely upregulated by IL4 and hypoxia; (2) DOR activation with specific DOR agonist UFP-512 significantly suppressed BV2 inflammatory transformations under LPS injury and hypoxic injury with high concentrations of UFP-512 being effective to inhibit the M2 transformations of microglia under IL4 condition; (3) DOR antagonism, naltrindole, enhanced both microglial M1 and M2 activation in LPS and hypoxic conditions; (4) DOR activation inhibited the production of inflammatory cytokines and iNOS under LPS injury and hypoxic injury, while reduced the secretion of anti-inflammatory cytokines under IL4 condition; (5) Knocking down DOR promoted microglia activation and inflammatory events, while overexpressing DOR induced an opposite effect; and (6) DOR-mediated protection on PC12 cells was enhanced by co-culturing with the conditioned medium from DOR agonist-treated BV2 cells under LPS insult or hypoxic stress.

Opioid receptors majorly comprise of three family members: μ-, δ- and κ-opioid receptors (MOR, DOR, and KOR). They are all G protein-coupled receptors and are widely distributed throughout the peripheral and central nervous system (Xia and Haddad, 1991, 2001; Xia, 2015; Machelska and Celik, 2020). Our previous studies have well-demonstrated that these opioid receptors play different roles in neural physiology and pathology with DOR being neuroprotective against hypoxic/ischemic insults and neurodegenerative injury (Shrivastava et al., 2017; Huang et al., 2018; Chen et al., 2020; Xu et al., 2020a). Little is known, however, about DOR distribution and function in microglia. Some previous studies examined MOR expression in glia, but there is little and contradictory data on DOR expression in glia. Some researchers showed that no DOR mRNA was detected by qPCR in microglia and astrocytes isolated from nucleus accumbent of rats (Schwarz et al., 2013). In sharp contrast, others observed prominent DOR expression in cultured N9 microglia, primary microglia and astrocytes isolated from rats (Turchan-Cholewo et al., 2008).

We provided the first evidence in this work that DOR maintained a low density in the BV2 cells, and its expression was closely associated with microglial dynamic polarization. Our data partially support the view of Cheng et al. (2021), who found a colocalization of DOR protein and CD68^+^ or F4/80^+^ labeled microglia in human and mouse brain tissues as well as an existence of DOR in BV2 culture cells. However, there is a major difference between our findings and those of Cheng’s in terms of the regulation of DOR expression in LPS-treated BV2 cells. They reported that BV2 cells significantly upregulated DOR expression after overnight exposure to 0.5 μg/ml LPS. In contrast, we found that LPS did not increase DOR expression at all, while IL4 and hypoxia remarkably upregulated DOR expression. Our results were based on the simultaneous comparison among LPS, IL4 and hypoxia groups and the consistency of both immunofluorescent and Western blot data. The reasons behind the difference between our observations and those of Cheng et al. (2021) are very likely related to the differences in the concentrations of LPS and the exposure durations between our and their experiments because DOR expression is sensitive to various stresses and differs in different conditions (Ma et al., 2005; Popiolek-Barczyk and Mika, 2016; Zhu et al., 2018; Polo et al., 2019; Xu et al., 2019).

We have previously shown that DOR expression is sensitively regulated in response to alterations in environmental condition (Ma et al., 2005; Zhang et al., 2006; Xia, 2015). For example, mild hypoxia, as a preconditioning stress, upregulates DOR expression, while severe hypoxia downregulates it with serious cell injury (Ma et al., 2005). In the present work, LPS might cause a relatively severe damage on the BV2 cells, thus leading to pathophysiological changes in the cells with a reduction in DOR density. On the contrary, IL4 could promote microglial M2 polarization, a phenotype for tissue repair and regeneration, and might mobilize protective strategies, including DOR upregulation, to protect the cells from injury.

Classical M1 phenotype and alternative M2 phenotype are two extremes of microglia activation, which is induced by LPS and IL4, respectively (Tang and Le, 2016). Although the concept of microglial M1/M2 polarization is not sufficient to describe the variety of microglial activation, M1/M2 classification and the relevant markers are still widely used in the research on microglia heterogeneity, especially for investigating the regulation of microglia-mediated inflammation (Devanney et al., 2020; Jiang et al., 2020; Liu et al., 2020; Saxena et al., 2021). Both clinical studies and bench investigations have shown that microglia are dynamically activated ranging between M1 and M2 phenotypes in response to different stages and severities of ischemic stroke (Hu et al., 2012; Kronenberg et al., 2018). Consistent with these findings, we have observed that hypoxic stress induced BV2 cells to transit toward M1 and M2 phenotypes as indicated by a significant increase of MHCII and arginase. Since M1-phenotype microglia expressed a low density of DOR (close to the control level) and M2-phenotype microglia expressed a high density of DOR, the fact that the level of DOR in the hypoxia group was in-between those of LPS and IL4 groups suggests that only partial BV2 cells, but not all of them, experienced M2 transformation.

Central nervous system inflammation plays a critical role in the progression of many cerebral diseases, and the inflammatory response is majorly dominated by microglia activation (Cunningham, 2013; Prinz et al., 2019; Azam et al., 2021). It is of high importance to explore novel agents to inhibit the excessive activations of microglia or eliminate the destructive shifts of microglia. Since DOR is sensitively regulated by environmental stresses in microglia, we speculated that DOR is a regulator in microglia activation and the relevant inflammatory events. Indeed, with multiple approaches, we found that the DOR activation with UFP-512, a specific DOR agonist, could significantly inhibit the microglial M1 shifts and suppressed the inflammatory events induced by the microglia transformations under LPS and hypoxic insults, while DOR inhibition enhanced the microglia M1 activation by upregulating MHCII. The opposite effects of DOR activation and inhibition on BV2 cells suggest a negative regulation of DOR in microglial activation and a close linkage between DOR and microglia-related inflammation.

It is noteworthy that high concentrations of UFP-512 could also inhibit the M2 shift of microglia, especially in the IL4-treated cells, while DOR inhibition using naltrindole could upregulate arginase, a M2 marker under LPS and hypoxic insults. In an earlier study, Mabrouk et al. (2009) reported the dual roles of DOR in Parkinsonian injury. They showed that administrations of lower doses (0.1–10 mg/kg) of UFP-512 attenuated the motor dysfunction of hemiparkinsonian rats, whereas higher doses (100–1,000 mg/kg) were ineffective or even worsened Parkinsonism. The same was true in the present study. Therefore, a proper concentration seems important for DOR’s beneficial effect against microglial inflammation. It is likely that DOR activation showed more beneficial effects in M1-microglia dominated environments, by inhibiting the inflammatory responses induced by various insults. Excessive amount of DOR agonists or DOR inhibition by naltrindole may bring complex outcomes. For example, although UFP-512 is a highly specific ligand for DOR, it still has a low binding affinity to MOR, or even KOR, the same is true for naltrindole. Therefore, a high concentration of UFP-512 may active MOR and/or KOR, and naltrindole may also inhibit MOR and KOR, inducing a complex effect. However, it is hard to completely rule out other possibilities at present. Nevertheless, our notion strongly suggests the importance of adequate concentrations of DOR agonists for targeting the regulation of microglial transformations.

To further clarify the role of DOR in the regulation of microglia, we directly manipulated DOR expression in the BV2 cells and then examining microglial states. By measuring several microglia activation markers, pro-inflammatory cytokines and anti-inflammatory cytokines, we concluded that DOR is critically required for anti-microglial activation. In the physiological condition, DOR knockdown significantly upregulated MHCII and Arginase, suggesting that DOR exerts a tonic inhibition of microglia activation. The fact that DOR knockdown increased the pro- and anti-inflammatory cytokines under LPS and hypoxia further demonstrated the importance of DOR in the negative control of microglia activation in stresses. With the same physiological condition, DOR overexpression showed a much milder effect on microglia activation. This is not surprised because the physiological level of endogenous DOR ligands might not be enough to fully activate all the overexpressed DOR. In fact, an over-reaction of DOR to microglia activation is unnecessarily needed in “normal” condition. In such a way, the cells avoid excessive energic costs and reserve the power of anti-microglial inflammation for emergency. The release of endogenous DOR ligand might increase in pathological condition, e.g., LPS insult and hypoxic stress. Under such stresses, the increased ligand bound to overexpressed DOR, thus leading to an amplifying DOR activity to suppress microglia activation and microglia induced inflammatory events.

Because of the unique role of the microglia in neuronal survival (Prinz et al., 2019; Azam et al., 2021), we further investigated if DOR-mediated alterations in microglia affect the cell viability of PC12 cells under stresses. Large amounts of documents have showed the supporting role of microglia for neurons by producing BDNF and IGF and regulating survival signaling pathways (Colonna and Butovsky, 2017; Wolf et al., 2017). Our data revealed that the medium from the BV2 cells treated by the DOR agonist or infected with DOR overexpression plasmid might have a considerable amount of pro-survival substances because they effectively increased the cell viability of PC12 against LPS and hypoxic insults. Moreover, we found that the neuroprotection via DOR-mediated regulation of microglia was more evident and appeared earlier under hypoxic stress, suggesting that the medium from the DOR agonist-treated BV2 cells or those with DOR overexpression had differential effects on the neurons in different stress states. This notion is also verified by the findings that the PC12 cells cultured in the medium from the naltrindole-treated BV2 cells or those with DOR knockdown always showed a decreased cell viability compared to the control under both LPS and hypoxic insults. However, it is interesting to note that the PC12 cells cultured in conditioned medium from naltrindole-treated BV2 cells also showed an increase in cell viability after 48-h LPS exposure. The exact mechanism is not known yet at this moment. One of the possibilities is that the DOR inhibition caused a feedback increase in the release of endogenous DOR ligand from the BV2 cells to the medium and the increased DOR ligand in the medium activated more DOR in the PC12 cells for neuroprotective signaling. However, this mechanism needs to be validated by further investigation because there is little information on the synthesis and release, even existence of endogenous DOR ligands in microglia (Turchan-Cholewo et al., 2008; Popiolek-Barczyk and Mika, 2016).

We initiated this work with the well-recognized M1/M2 markers and focused on the role of DOR in microglia-related inflammatory events. As we discussed above, however, there is growing appreciation that the concept of M1/M2 classification largely oversimplifies the complexity of microglia phenotypes, especially for *in vivo* functions (Devanney et al., 2020). Because there is no sufficient data in our hands regarding the complex dynamic changes in microglia under IL4/LPS/hypoxia, we do not want to over- speculate the role of DOR in other microglial phenotypes at this moment. However, the in-depth investigation into the effects of DOR on microglia, beyond microglial M1/M2 polarization, are required in future.

In summary, we present the first data to demonstrate a critical role of DOR in the regulation of microglia polarizations. DOR activation could effectively suppress the inflammatory shift of the microglia under LPS insult and hypoxic stress, while DOR inhibition or DOR knockdown promoted microglia activation. Although hypoxic stress induced biphasic activation of BV2 cells, the microglia-relevant pro-inflammatory activities seem stronger as indicated by a more evident increase in iNOS, CD86, and TNF-α. Specific DOR activation or DOR overexpression dominantly suppressed the destructive activation of microglia, and promoted the neuronal viability under hypoxic injury. Together with the evidence that excessive microglia activation is implicated in the pathogenesis of various neurodegenerative diseases, and microglia activation is evident in the aged brains (Wu et al., 2020), our findings suggest that DOR is an effective protector against neurodegenerative pathophysiology by modulating microglia activities.

## Data Availability Statement

The datasets presented in this study can be found in online repositories. The names of the repositories and the raw data link of this article will be made available, by the authors, to the qualified researchers upon completion of the research request form.

## Author Contributions

YuX, FZ, and YiX conceived and designed the experiments. YuX and JM performed the experiments and analyzed the data. YuX, YY, YP, GB, and YiX contributed reagents/materials/analysis tools. YiX and FZ supervised the conduct of the work. YuX and YiX wrote the manuscript. All authors read and approved the final manuscript.

## Conflict of Interest

The authors declare that the research was conducted in the absence of any commercial or financial relationships that could be construed as a potential conflict of interest.

## Publisher’s Note

All claims expressed in this article are solely those of the authors and do not necessarily represent those of their affiliated organizations, or those of the publisher, the editors and the reviewers. Any product that may be evaluated in this article, or claim that may be made by its manufacturer, is not guaranteed or endorsed by the publisher.
